# Prevalence of obesity, hypertension and diabetes among people living with HIV in South Africa: a systematic review and meta-analysis

**DOI:** 10.1186/s12879-023-08736-5

**Published:** 2023-12-07

**Authors:** Jacob M. Gizamba, Jess Davies, Chad Africa, Candice Choo-Kang, Julia H. Goedecke, Hlengiwe Madlala, Estelle V. Lambert, Dale E. Rae, Landon Myer, Amy Luke, Lara R. Dugas

**Affiliations:** 1 Division of Epidemiology and Biostatistics, School of Public Health, University of Cape Town, Cape Town, South Africa; 2 Spatial Science Institute, University of Southern California, Los Angeles, USA; 3 Public Health Sciences, Parkinson School of Health Sciences and Public Health, Loyola University Chicago, Maywood, IL USA; 4 Biomedical Research and Innovation Platform, South African Medical Research Council, Tygerberg, Cape Town, South Africa; 5 Health Through Physical Activity, Lifestyle and Sport Research Centre, Division of Physiological Sciences, Faculty of Health Sciences, University of Cape Town, Cape Town, South Africa

**Keywords:** People living with HIV, Obesity, Hypertension, Diabetes, Meta-analysis, Prevalence

## Abstract

**Background:**

HIV has become a manageable chronic condition due to the success and scale-up of antiretroviral therapy (ART). Globally, South Africa has the highest number of people living with HIV (PLHIV) and research evidence indicates that countries with the highest burden of PLHIV have a substantial burden of obesity, hypertension (HPT) and type 2 diabetes (T2D). We sought to summarize the burden of these three common NCDs among PLHIV in South Africa.

**Methods:**

In this systematic review, multiple databases were searched for articles reporting on the prevalence of obesity, HPT, and T2D among PLHIV in South Africa published since journal inception until March 2022. A meta-analysis was conducted using random-effects models to obtain pooled prevalence estimates of the three NCDs. Heterogeneity was assessed using X^2^ test on Cochran’s Q statistic.

**Results:**

We included 32 studies, with 19, 22 and 18 studies reporting the prevalence of obesity, HPT, and T2D among PLHIV, respectively. The overall prevalence of obesity, HPT, and T2D was 23.2% [95% CI 17.6; 29.9], 25.5% [95% CI 15.6; 38.7], and 6.1% [95% CI 3.8; 9.7] respectively. The prevalence of obesity was significantly higher among women (*P* = 0.034) compared to men, however the prevalence of HPT and T2D did not differ by sex. The prevalence of each of the three NCDs did not differ significantly between rural, urban, and peri-urban areas. The prevalence of obesity and T2D was higher in studies conducted between 2013 and 2022 compared to studies conducted between 2000 and 2012, while the prevalence of HPT was higher between 2000 and 2012 compared to between 2013 and 2022.

**Conclusions:**

These findings suggest that South Africa is experiencing a syndemic of NCDs among people PLHIV highlighting the need to increase cost-effective interventions and management strategies that involve integrated HIV and NCD care in the South African setting.

**Supplementary Information:**

The online version contains supplementary material available at 10.1186/s12879-023-08736-5.

## Introduction

Human Immunodeficiency Virus (HIV) is a global public health threat with approximately 40 million people living with HIV (PLHIV). Sub-Saharan Africa (SSA) is the most affected region and South Africa has the highest number of PLHIV (approximately 7.7 million) [1]. Currently HIV has become a manageable chronic condition due to the success and scale-up of antiretroviral therapy (ART) and South Africa has been reported to have the largest ART program globally, with approximately 4.6 million people on treatment [2–6]. Increased life expectancy among PLHIV has become associated with the concurrent increase in the prevalence of non-communicable diseases (NCDs) such as obesity, hypertension (HPT) and type 2 diabetes (T2D) [7, 8]. There is a co-existence of both chronic infectious and non-infectious diseases, both exacerbated by a highly unequal society, poverty and other social determinants, and which reflect an epidemiological transition [1, 9].

The prevalence of obesity is increasing among adults in SSA, with South Africa recording the highest obesity rates, especially among women [10, 11]. Notably, the prevalence of overweight and obesity in South Africa is 67.5% among women and 31.3% among men who are 15 years and above according to the 2016 SA demographic health survey (SADHS) [12]. This has been accompanied by dramatic increases in the prevalence of HPT and T2D [13, 14]. By way of illustration, from 1998 to 2008, the age-adjusted prevalence of HPT increased by more than 10% (rising from 24.4 to 35.1% among both men and women aged 15 years and above) [15]. Furthermore, according to the 2016 SADHS data, the HPT prevalence in women was 46.0% and 44.0% in men [12]. The projected rates of T2D increase (150%) are highest in SSA compared to other International Diabetes Federation (IDF) regions [16], significantly increasing the burden of NCDs in the region. Further, South Africa has the highest number of people with T2D [17] and T2D is the second leading cause of death in the country and the leading cause of death among women [18]. The IDF projects that over 8 million South African adults will be living with T2D by 2045 [16].

Due to the already high prevalence of HIV, obesity, HPT and T2D in the general population of South Africa, there is a need to understand the extent to which the prevalence of these three common NCDs may differ among PLHIV within the South African context. Additionally, because studies comparing rural and urban areas of South Africa have reported different burdens of obesity, HPT and T2D in the general population [19–21], it was important to determine if these same patterns in prevalence of NCDs are seen in PLHIV. The juxtaposition of chronic infectious and NCDs in such a context is particularly relevant and could highlight the need for scaling of the South African national government’s plan for integrated chronic disease management.

Therefore, we performed a systematic review and meta-analysis to explore the prevalence of obesity, HPT and T2D among PLHIV in South Africa, in an era of increased life-expectancy among PLHIV. A sub aim was to explore differences in the prevalence by setting (i.e., urban, rural, and peri-urban areas), sex and by year of the study.

## Methods

### Study design

The systematic review and meta-analysis were guided and reported in accordance with the MOOSE guidelines [22]. We searched for articles in Scopus, Cochrane library, PubMed, Google Scholar, and Web of Science that reported the prevalence of obesity, HPT, and T2D among PLHIV in South Africa published since journal inception until March 2022.

### Eligibility criteria

Literature that used objectively measured prevalence estimates of either obesity, HPT or high blood pressure, or T2D among PLHIV within South Africa were included. Studies that used self-report for HPT and T2D were included as well. Peer reviewed journals were not excluded by year of publication or language, but studies must have used human participants. Studies were included irrespective of the study design used (i.e., randomized control trials (RCTs) and observational studies). Papers were excluded if they were qualitative studies, modelling studies, involved pregnant and postpartum women, children, and conducted outside South Africa.

### Data collection procedures

#### Search strategy

A search strategy that employed medical subject headings (MeSH) and keywords was developed and used while searching for literature from all the selected databases. The following key terms were used in the search strategy for all the databases; “South Africa”, “HIV”, “AIDs”, “Epidemiology”, “Prevalence”, “HPT”, “high blood pressure”, “T2D”, “diabetes”, “obese”, and “obesity”. The Boolean terms (AND, OR) were used to separate keywords, and MeSH was conducted in advanced searching of articles. The final search strategy for PubMed can be found in Supplementary Table 1. The same approach was adopted for other databases. A secondary search of relevant articles from reference lists of included studies was also undertaken.

### Selection of sources of evidence

Retrieved articles were uploaded into EndNote to remove duplicates. The articles were then uploaded to Rayyan QCRI (Copenhagen: The Nordic Cochrane Centre, Cochrane) [23] where they were independently screened based on title and abstract by two reviewers (JMG, CA). The screening process was done following the eligibility criteria. Then full texts of studies that passed the initial stage of screening were retrieved and screened to verify their conformance with the inclusion criteria. Disagreements on selected studies were discussed and resolved by consensus or the intervention of a third reviewer when necessary.

### Statistical methods

#### Data extraction process

For all included studies, three reviewers (JMG, CA and JD) extracted the data using a data abstraction tool which the authors designed purposely for this study. The following information was extracted: Name of first author and year of publication, study objective, study location (province, rural/urban, site setting), study population (sample size, age), study design, data collection period, NCD studied, diagnostic method, ART status (initiated vs uninitiated), NCD prevalence estimates or data that could be used to self-calculate the prevalence of NCDs among PLHIV. Any disagreements were resolved through discussion between the two reviewers or further adjudication by a third reviewer.

### Risk of Bias and quality of included studies

Two reviewers evaluated the methodological quality and risk of bias of included studies using the Risk of Bias tool for prevalence studies developed by Hoy et al. [24].

### Synthesizing the results

Data were analyzed with R software (version 3.6.1) using the meta package. Forest plots were drawn to visualize the pooled prevalence and the 95% confidence intervals (CI) of obesity, HPT, and T2D among PLHIV in South Africa and the extent of heterogeneity between studies. Results from pregnant women were excluded while estimating the pooled prevalence of each NCD. A random effects meta-analysis model based on the DErSimonian-Laird inverse-variance method and the Freeman-Tukey double arc-sine transformation for proportions was used to pool the prevalence data because of the inherent differences between the participants in each study [25]. The robustness of the pooled crude prevalence estimate was assessed by doing sensitivity analysis including only studies that had a low risk of bias.

Heterogeneity was assessed using the χ2 test on Cochrane’s Q statistic and quantified by calculating the I^2^ (with values of 25%, 50% and 75% being representative of low, medium, and high heterogeneity, respectively). Because of the high degree of heterogeneity, subgroup analyses were conducted using the study setting (rural, urban, and peri urban), year in which the study was conducted, sex, diagnostic criteria, and ART status. To assess publication bias, a funnel plot and Egger weighted regression methods were used and a *p*-value < 0.05 was considered indicative of statistically significant publication bias. Agreement between reviewers for study inclusion, data extraction and risk of bias assessment was assessed using the Cohen’s Kappa coefficient.

## Results

### Selection of sources of evidence

**Fig. 1:**
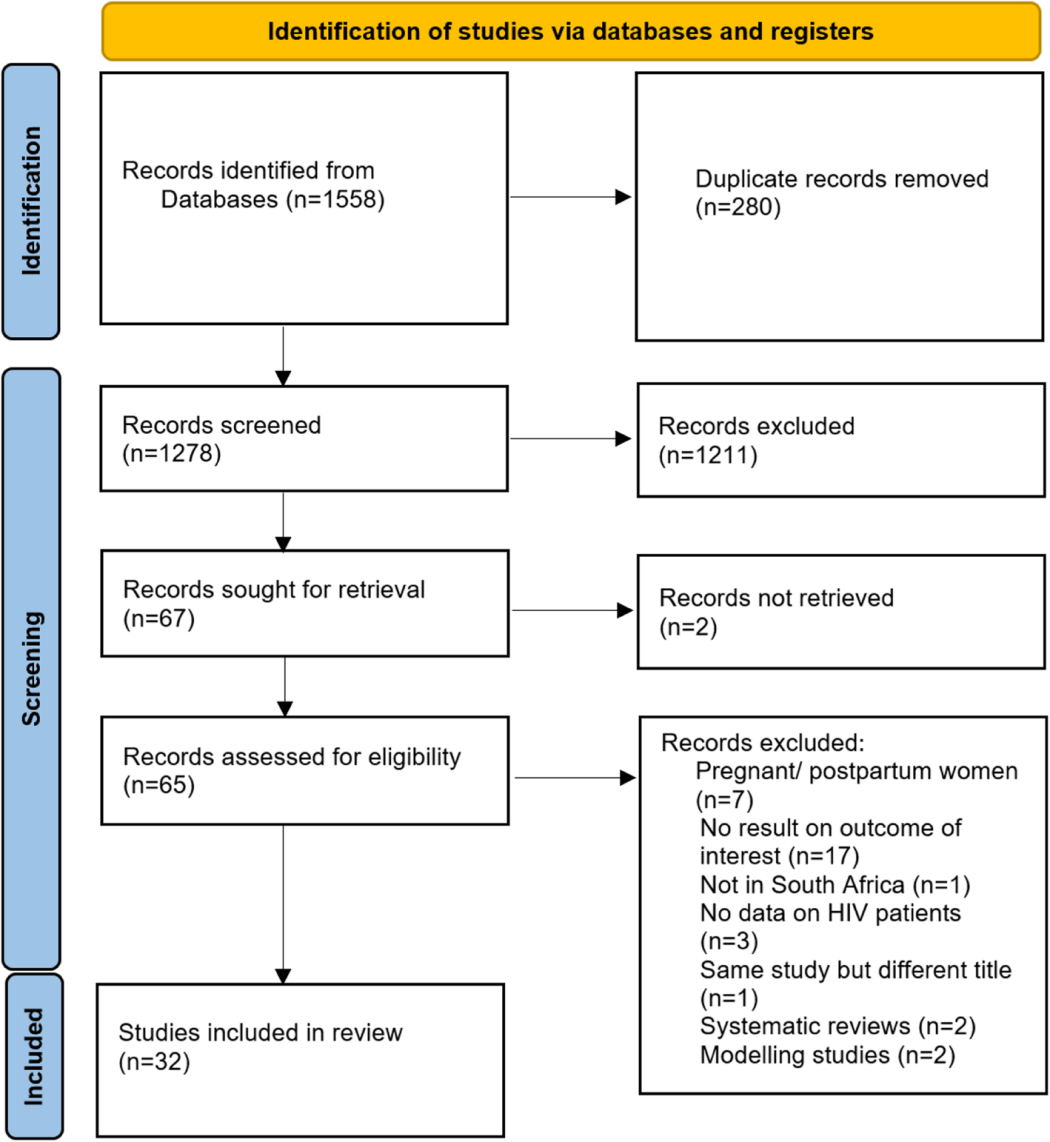
Flow diagram depicting selection of included studies

We found a total of 1,558 articles after the search. After removing 280 duplicates, we screened the titles and abstracts of 1,278 articles and excluded 1,211 that did not meet our inclusion criteria. We then reviewed the full texts of 65 articles and excluded 33 articles for the following reasons: 7 articles involved pregnant or postpartum women; 17 articles had no results on outcomes of interest; 1 article was not done in South Africa; 3 articles did not report results for HIV participants; 1 article was a duplicate but with a different title; 2 articles were systematic reviews because relevant articles contained in these reviews had already been selected for inclusion; and 2 articles were modelling studies. The full text of two articles were not accessible. In total, 32 studies that reported prevalence estimates for obesity, HPT and T2D among PLHIV in South Africa were reviewed (Fig. 1).

### Characteristics of sources of evidence

The characteristics of the included studies; the province, study setting, study design, sample size, population sample age and sex and the risk of bias for each study are presented in Table 1. The studies were published between 2004 and 2020, although 66.7% of the studies were published after 2012. Of the 32 studies, 22 studies used a cross-sectional design, 6 were cohort studies, and 4 were national and community-based surveys. The majority of the studies (*n* = 9, 28.1%) were conducted in Western Cape (WC), KwaZulu Natal (KZN, *n* = 9, 28.1%) and Gauteng (GA, *n* = 5, 15.6%), with less representation of the Eastern Cape (EC), Free State (FS), Mpumalanga (MP) and Limpopo (LM) provinces**.** No studies were included from Northern Cape and Northwest provinces. The majority (*n* = 20, 62.3%) of studies were conducted in health care facility settings and the remainder were community-based studies. In terms of study setting, most of the studies (*n* = 15, 46.9%) were conducted in urban areas, followed by 6 (18.8%) studies in peri-urban townships and only 5 (15.6%) studies were conducted in a rural area, while 6 (18.8%) studies were conducted both in urban and rural areas. Out of the 32 studies, 5 were conducted among women only, 26 studies included both men and women, and 1 study included only men. Most studies (*n* = 30) were conducted amongst adults (18 years and older), and 1 study included participants who were 15 years and older, and 1 study did not report the age range of the participants. Of the included studies, 18 (56.3%) involved participants that were on ART, 10 (31.3%) studies involved participants who were not on ART or who initiated ART during the study, 1 study included both ART initiated and uninitiated participants while 3 studies did not report the ART status of included participants. Among the included studies, 19 (59.4%), 22 (68.8%), and 18 (56.3%) reported prevalence rates for obesity, HPT, and T2D, respectively. Twelve studies exclusively examined one of the NCDs, 14 studies investigated two NCDs, and 6 studies investigated all three NCDs simultaneously.

**Table 1:** Summary characteristics of the studies included

Province	First author published year	Study setting	Study area	Study design	Study period	Sample size	Sex	ROB
**Western Cape**	Abrahams 2015 [26]	ART clinics	Urban	cohort study	2015	103	Women only	low
	Borkum 2017 [27]	Crossroads Community Health Centre	Urban	cross-sectional study	2017	67	Women and Men	moderate
	Gausi 2021 [28]	Integrated clubs primary care clinics	Urban	retrospective cohort study	2005	247	Men only	low
	George 2019 [29]	Khayelitsha, ubuntu ART clinic	Peri-Urban Township	cross-sectional study	2015	330	Women only	moderate
	Hyle 2019 [30]	Cape town	Peri-Urban Township	cross‐sectional study	2016	458	Women and Men	low
	Levitt 2016 [31]	Community health care centre	Urban	community-based survey	2010	880	Women and Men	moderate
	Mutemwa 2018 [32]	Public healthcare facilities	Urban and Rural	cross-sectional survey	2015	833	Women and Men	low
	Oni 2017 [33]	TB clinic in Cape Town	Peri-Urban Township	cross sectional		852	Women and Men	low
	Oni 2015 [34]	Michael Mapongwana clinic, PHC in Khayelitsha	Peri-Urban Township	cross sectional study	2013	14,364	Women and Men	low
**KwaZulu-Natal**	Barnighausen 2008 [35]	rural Umkhanyakude	Rural	large population-based survey	2004	3574	Women only	low
	Biggs 2017 [36]	Lancers Road Clinic	Urban	cross-sectional study	2013	84	Women and Men	moderate
	Naidoo 2018 [37]	KwaZulu-Natal	Urban and Rural	retrospective cohort study	2010	948	Women and Men	low
	Hanley 2021 [38]	(PHC) clinics in Umlazi	Peri-Urban Township	cross sectional analysis	2019	372	Women only	low
	Hurley 2011 [39]	Sinikithemba HIV Clinic at McCord Hospital in Durban	Urban	observational, descriptive cohort study design	2009	230	Women and Men	low
	Malaza 2012 [40]	rural Umkhanyakude	Rural	population-based survey	2010	14,918	Women and Men	low
	Mohammed Umar 2021 [41]	4 HIV clinics at Public Sector Hospitals in the eThekwini	Urban	retrospective cohort study	2019	1203	Women and Men	low
	van Heerden 2017 [8]	Home based	Rural	cross sectional:	2012	570	Women and Men	low
	Rajagopaul 2021 [42]	Wentworth Hospital, a district facility in Durban	Urban	cross sectional study	2016	301	Women and Men	moderate
**Gauteng**	Hopkins 2019 [43]	HIV testing services centre in Soweto	Urban	cross sectional	2018	325	Women and Men	low
	Hopkins 2021 [44]	HIV testing services centre in Soweto	Urban	cross sectional	2019	780	Women and Men	low
	Julius 2011 [45]	HIV clinic	Urban	cross sectional	2009	304	Women and Men	moderate
	Mahlangu 2020 [46]	Tshwane Health District	Urban	cross sectional	2018	480	Women and Men	moderate
	Mashinya 2014 [47]	Dikgale Health and Demographic Surveillance System Centre	Rural	cross sectional study nested in case control study	2012	267	Women and Men	low
**Mpumalanga**	Mathebula 2020 [48]	Rixile ART Clinic, Tintswalo Hospital	Peri-Urban Township	cross sectional study	2020	332	Women and Men	low
**Free State**	Rabkin 2015 [49]	Community Health Centre	Urban	cross-sectional study	2014	175	Women and Men	moderate
	Hattingh 2011 [50]	the city of Bloemfontein	Urban	cross sectional		500	Women only	low
**Eastern Cape**	Sogbanmu 2019 [51]	Buffalo City Municipality, East London	Urban	cross sectional study	2017	335	Women and Men	low
**Limpopo**	Wensink 2015 [52]	Rural PHC, Ndlovu Medical Centre	Rural	cross-sectional study	2013	903	Women and Men	low
**All provinces**	Zungu 2019 [53]	all provinces	Urban and Rural	Cross-sectional	2012	21,496	Women and Men	low
	Brennan 2018 [54]	public-sector clinics	Urban and Rural	observational cohort study	2017	77,696	Women and Men	low
	Chiweenie 2021 [55]	national study	Urban and Rural	cross-sectional population-based survey	2005	978	Women and Men	low
	Godongwana 2021 [56]	9 provinces of South Africa	Urban and Rural	retrospective cohort analysis	2010	95,701	Women and Men	moderate

*ART* Anti-retroviral treatment, *TB* Tuberculosis, *PHC* Primary Healthcare Centre

### Prevalence of obesity among PLHIV in South Africa

**Fig. 2:**
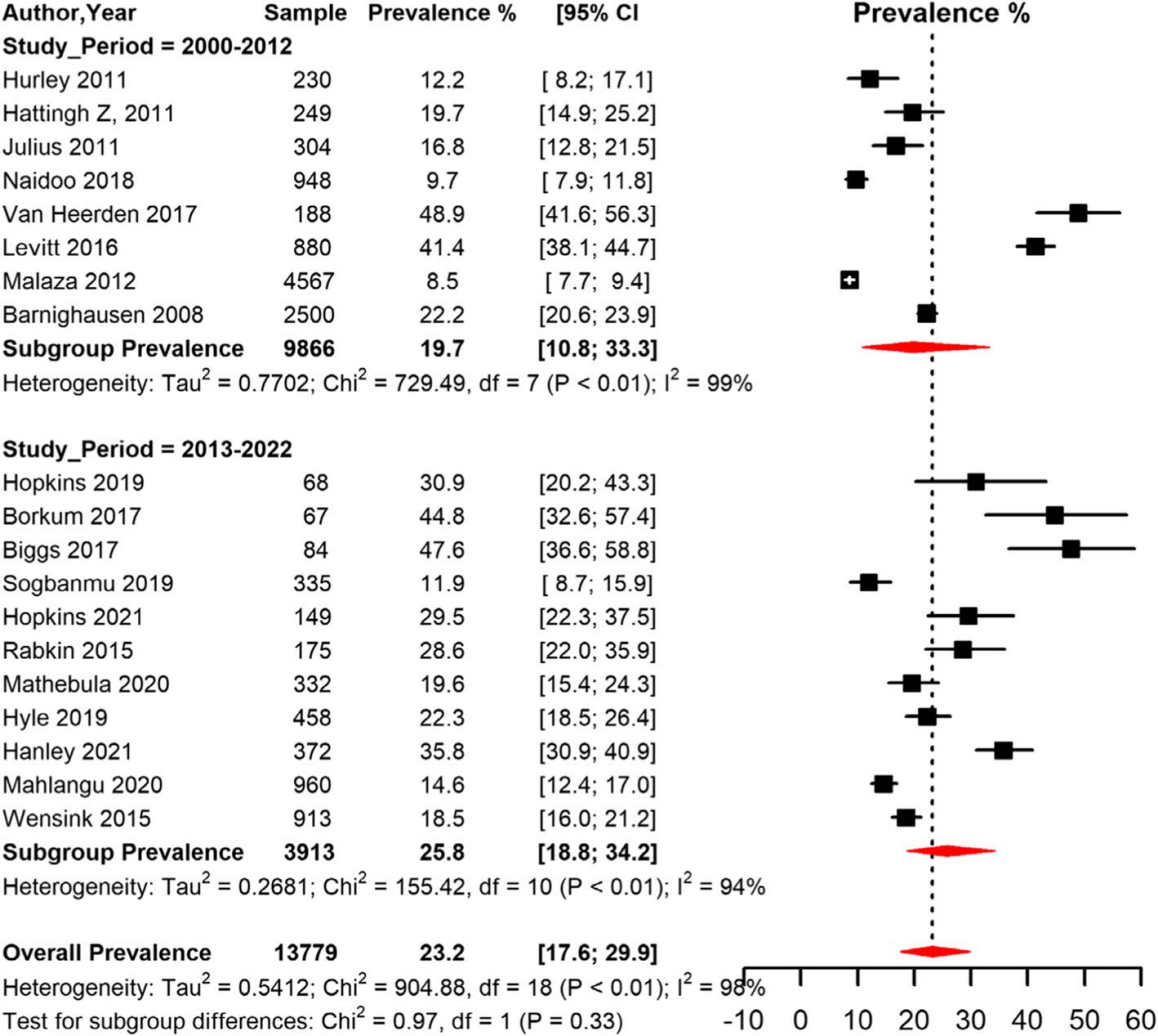
Meta-analysis prevalence of obesity among people living with HIV in South Africa grouped by study period (2000–2012 and 2013–2022) CI: confidence interval. The vertical dotted line represents the overall prevalence, and the red diagonals represent the pooled prevalence in each group

Overall, data from 13,779 PLHIV from 19 studies that reported prevalence of obesity were included. All 19 studies used the WHO criteria as reference for body mass index (BMI) cut-off values for obesity [57]. Most of the studies that reported the prevalence of obesity were conducted in urban (*n* = 11) settings followed by rural (*n* = 4), peri-urban (*n* = 3) settings and one study was conducted both in urban and rural settings. Meta-analysis data indicate that the overall prevalence of obesity in South Africa among PLHIV was 23.2% [95% CI 17.6; 29.9] with high heterogeneity (I^2^ = 99.0%, *p* < 0.001) (Table 2, Fig. 2). Based on the funnel plot (Supplementary Fig. 1) and Egger’s test, there was symmetry and no evidence of potential publication bias (*p* = 0.202) for determining the prevalence of obesity among PLHIV. The sensitivity analysis including only low risk of bias studies yielded a slightly lower prevalence estimate to that of the crude analysis (Table 2).

**Table 2:** Meta-analysis prevalence of obesity among people living with HIV in South Africa

	**prevalence (95% CI)**	**N studies**	**N participants**	**sub-group p-value**
Global ^Ϣ^	23.2 (17.58;29.89)	19	13,779	
low ROB ^β^	20.3 (14.50;27.77)	13	11,309	
By sex				
Men	12.1 (6.27;22.09)	15	5,036	0.034
Women	23.6 (17.41;31.18)	17	8,608	
By study period				
2000–2012	19.7 (10.83;33.25)	8	9,866	0.325
2013–2022	25.8 (18.78;34.24)	11	3,913	
By setting				
Rural	21.5 (5.64;55.61)	4	8,168	0.919
Urban	25.0 (17.26;34.80)	11	3,501	
Peri-urban	25.4 (10.26;50.27)	3	1,162	
ART status ^ϱ^				
initiated	25.9 (19.16;33.90)	10	4,610	0.653
uninitiated	22.7 (11.12;40.69)	7	2,102	

*ART* Anti-retroviral therapy, *CI* Confidence interval, *ROB* Risk of bias

Ϣ overall pooled prevalence

β pooled prevalence with only studies with a low ROB

ϱ Initiated means participants were already on ART at time of enrollment into the study while uninitiated means participants were not on ART or initiated ART at start or during course of the study

**Fig. 3:**
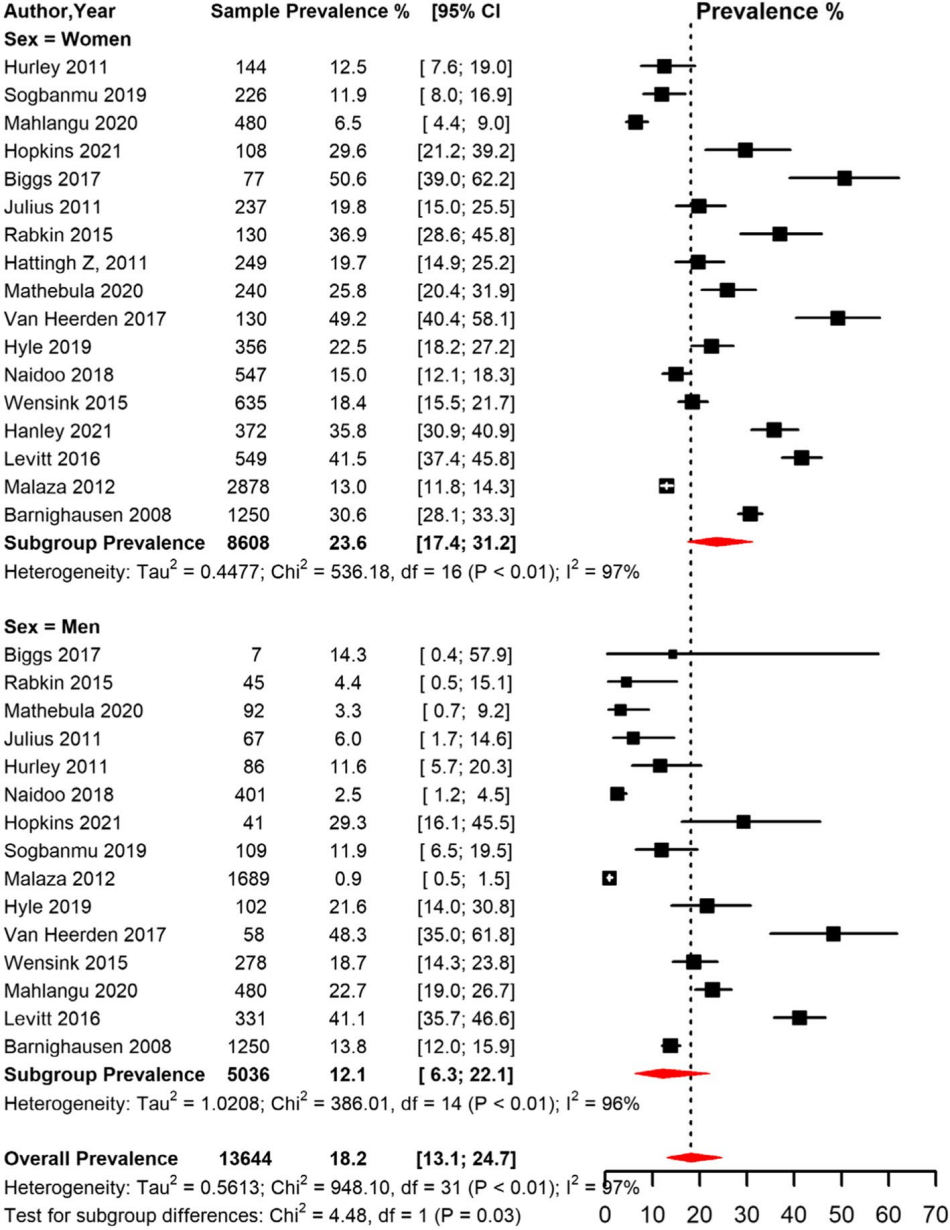
Meta-analysis prevalence of obesity among people living with HIV in South Africa grouped by sex.CI: confidence interval. The vertical dotted line represents the overall prevalence, and the red diagonals represent the pooled prevalence for each sex

**Fig. 4:**
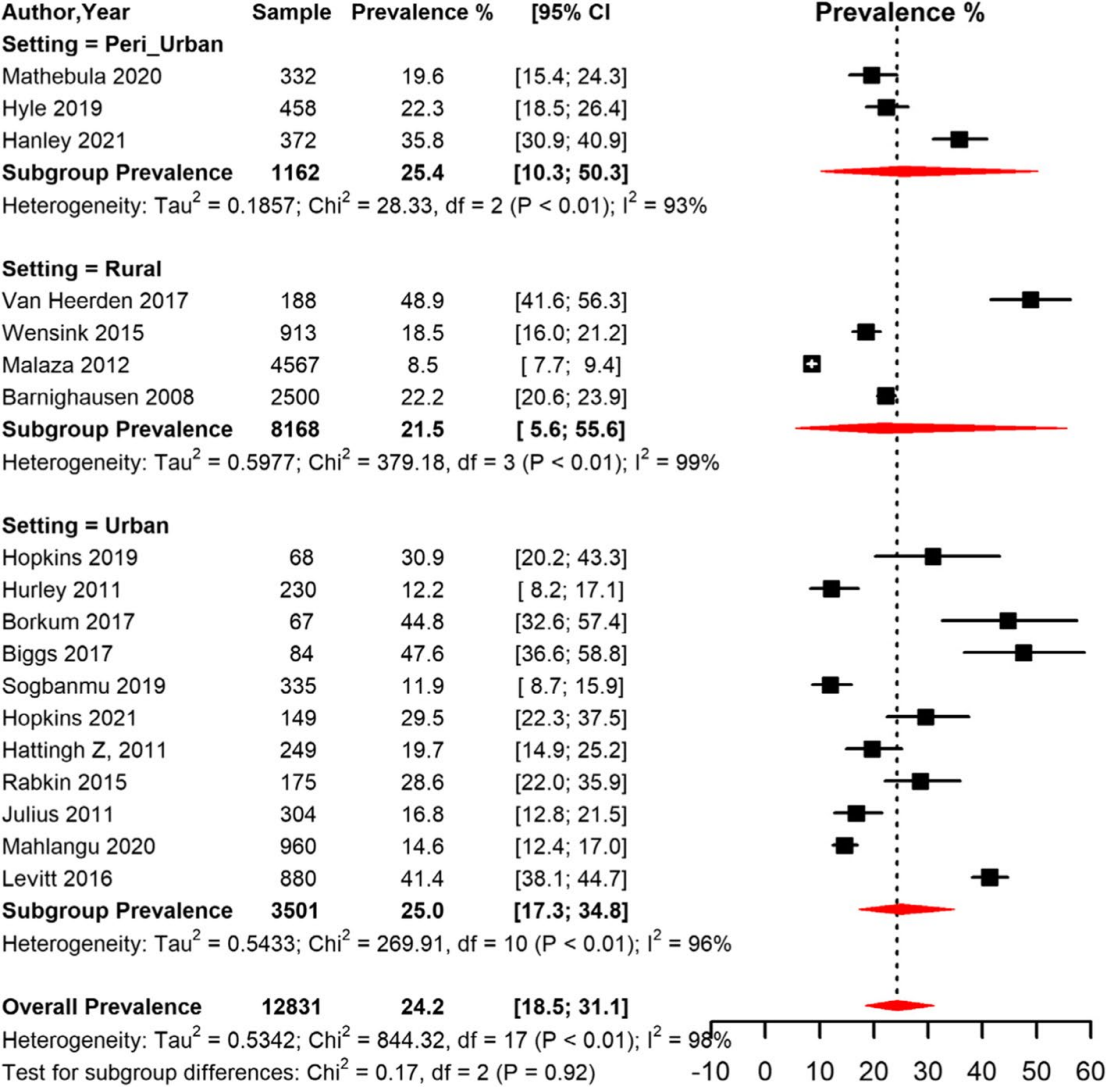
Meta-analysis prevalence of obesity among people living with HIV in South Africa grouped by setting (Urban, peri-urban, and rural areas) CI: confidence interval. The vertical dotted line represents the overall prevalence, and the red diagonals represent the pooled prevalence in each setting

The prevalence of obesity was significantly higher (*p* = 0.034) in women (23.6% [95% CI 17.4; 31.2]) compared to men (12.1% [95% CI 6.3; 22.1]) (Table 2, Fig. 3). The pooled prevalence was higher in studies conducted in peri-urban (25.4% [95% CI 10.3; 50.3]) and urban (25.0% [95% CI 17.3; 34.8]) compared to rural areas (21.5% [95% CI 5.6; 55.6]) (Table 2, Fig. 4). The prevalence was higher in studies conducted between 2013–2022 (25.8% [95% CI 18.8; 34.2]) compared to studies conducted between 2000–2012 (19.7 [95% CI 10.8; 33.3]) (Fig. 2). The prevalence was also higher in studies conducted among participants who were on ART (25.9% [95% CI 19.2; 33.9]) compared to studies conducted among participants not initiated on ART (22.7 [95% CI 11.1; 40.7]) (Table 2).

### Prevalence of HPT among PLHIV in South Africa

In total, data from 123,951 PLHIV from 22 studies that reported prevalence of HPT were included. Different diagnostic criteria were utilised to categorise participants as hypertensive or normotensive. These included: participant self‐reported history of HPT diagnosis by a professional physician (*n* = 1); self-reported use of antihypertensive drugs (*n* = 1), blood pressure (BP) measurements (*n* = 11) whereby a participant with systolic BP readings of ≥ 140 mmHg and/or diastolic BP ≥ 90 mmHg was considered hypertensive; either BP measurement or self-reported use of antihypertensive drugs (*n* = 8); either BP measurement or self-reported history of hypertension (*n* = 1).

**Fig. 5:**
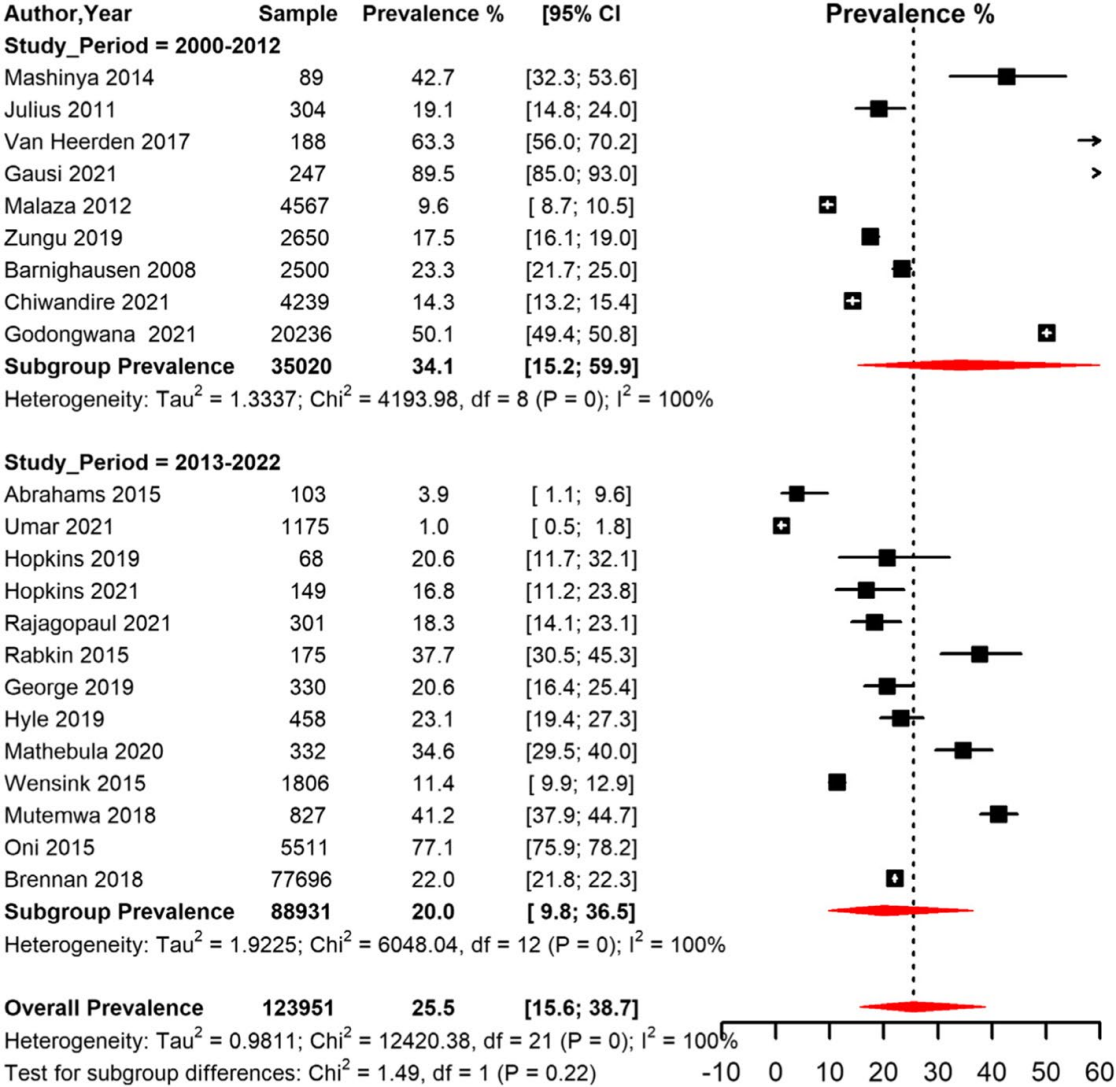
Meta-analysis of prevalence of Hypertension among people living with HIV in South Africa grouped by study period (2000–2012 and 2013–2022) CI: confidence interval. The vertical dotted line represents the overall prevalence, and the red diagonals represent the pooled prevalence in each group

Overall, the prevalence of HPT among PLHIV in South Africa was 25.5% [95% CI 15.6; 38.7], and the heterogeneity between the studies was high (I2 = 99.0%, *p* < 0.001) (Table 3, Fig. 5). The Egger’s test and the funnel plot suggested no evidence of publication bias (*p* = 0.937) (Supplementary Fig. 2). The sensitivity analysis including only low risk of bias studies yielded a similar prevalence estimate to that of the crude analysis (Table 3).

**Table 3:** Meta-analysis prevalence of Hypertension among people living with HIV in South Africa

	**prevalence (95% CI)**	**N studies**	**N participants**	**sub-group *p*-value**
**Global** ^Ϣ^	25.5 (15.59;38.72)	22	123,951	
**low ROB**^β^	24.7 (12.84;42.20)	17	102,605	
**By sex**				
** Men**	22.4 (11.66;38.57)	19	49,117	0.631
** Women**	27.0 (14.79;44.00)	20	74,766	
**By study period**				
** 2000–2012**	34.1 (15.19;59.92)	9	35,020	0.223
** 2013–2022**	20.0 (9.78;36.49)	13	88,931	
**By setting**				
** Rural**	25.6 (7.38;59.85)	5	9,150	0.536
** Urban**	18.5 (4.27;53.68)	8	2,522	
** Peri-urban**	38.0 (8.58;80.0)	4	6,631	
**Diagnostic criteria**				
** BP measurement**	26.7 (11.48;50.47)	12	34,701	0.610
** BP measurement/HPT drugs** ^**ɞ**^	20.7 (12.22;32.86)	8	81,089	
** Self-report**	45.8 (0.00;100.00)	2	8,161	
**ART status** ^ϱ^				
**initiated**	24.2 (11.08;44.91)	14	89,414	0.459
**uninitiated**	34.1(9.84;71.05)	4	2,995	

*ART *Anti-retroviral therapy, *BP *Blood pressure, *CI *Confidence interval, *HPT *Hypertension, *ROB *Risk of bias

Ϣ overall pooled prevalence

β pooled prevalence with only studies with a low ROB

ɞ Blood pressure measurement or self-report of use of antihypertensive drugs

ϱ Initiated means participants were already on ART at time of enrollment into the study while uninitiated means participants were not on ART or initiated ART at start or during course of the study

**Fig. 6:**
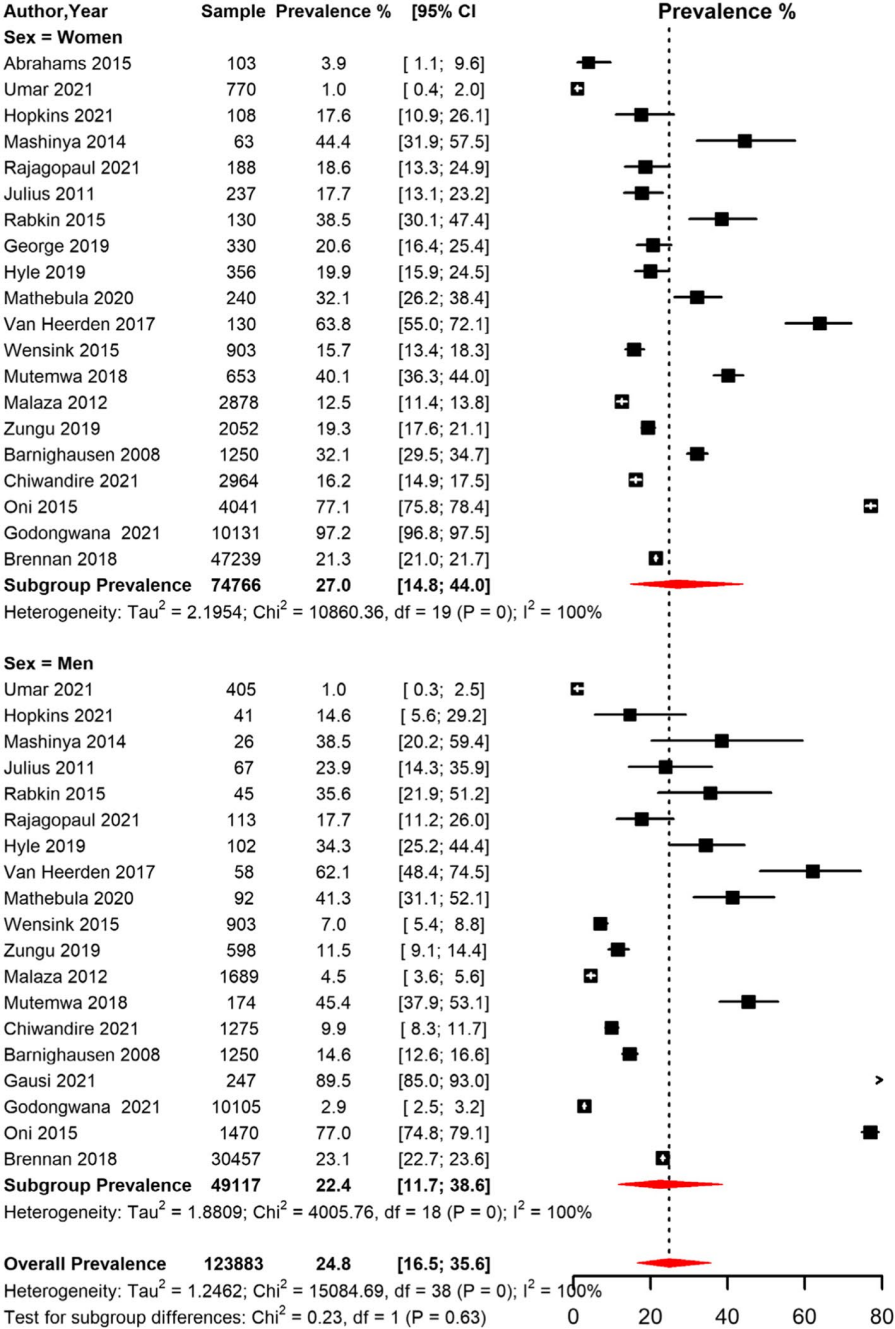
Meta-analysis of prevalence of Hypertension among people living with HIV in South Africa grouped by sex. CI: confidence interval. The vertical dotted line represents the overall prevalence, and the red diagonals represent the pooled prevalence for each sex

**Fig. 7:**
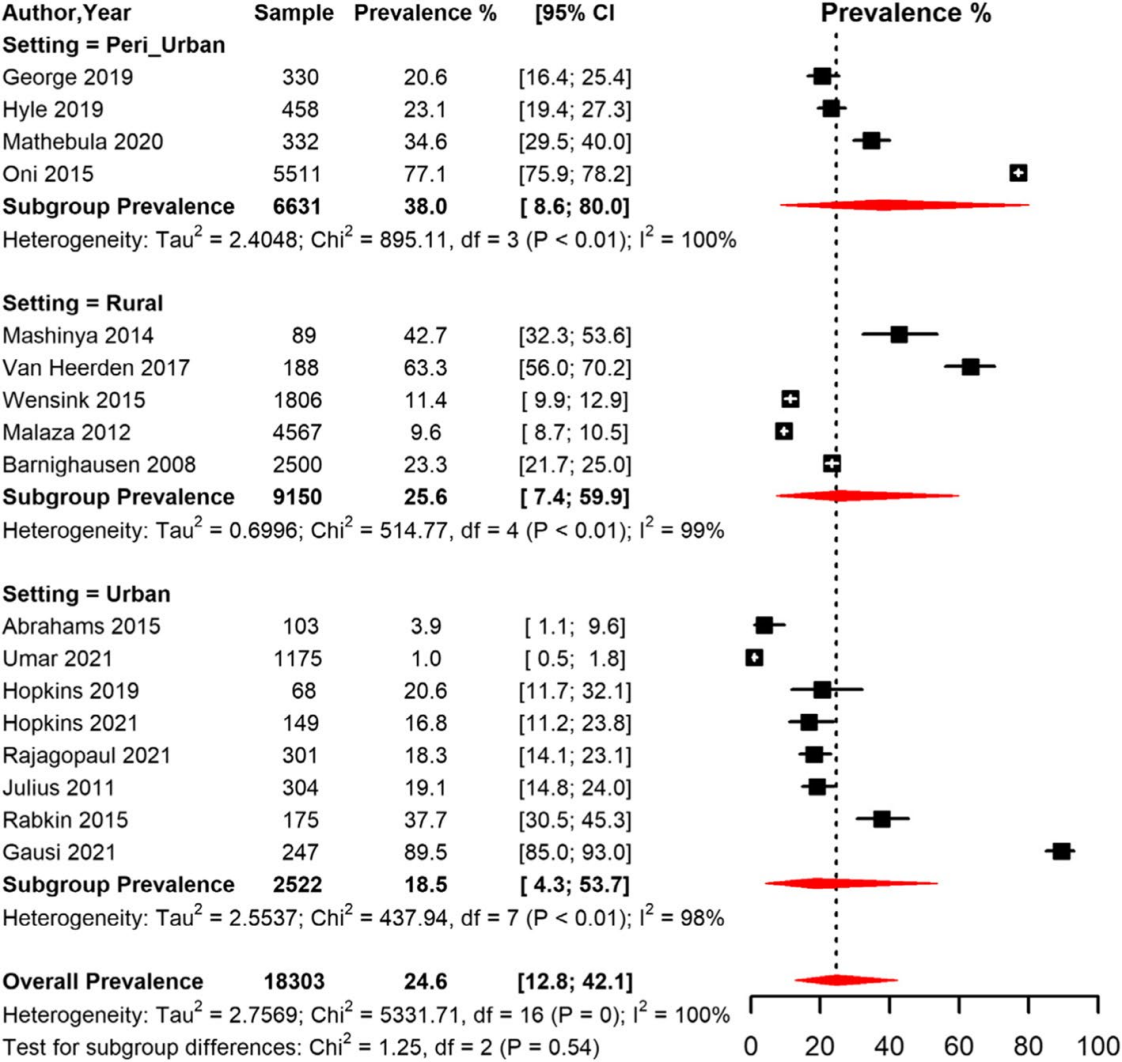
Meta-analysis of prevalence of Hypertension among people living with HIV in South Africa grouped by setting (rural, peri-urban, and urban areas) CI: confidence interval. The vertical dotted line represents the overall prevalence, and the red diagonals represent the pooled prevalence in each setting

The pooled prevalence of HPT among men and women was 22.4% [95% CI 11.7;38.6] and 27.0% [95% CI 14.8;44.0] respectively (Table 3, Fig. 6). The pooled prevalence for studies conducted in rural areas was 25.6% [95% CI 7.4; 59.9]), peri-urban areas 38.0% [95% CI 8.6; 80.0] and urban areas 18.5% [95% CI 4.3;53.7]) (Table 3, Fig. 7). The prevalence was lower in studies conducted among participants who were on ART (24.2% [95% CI 11.1; 44.9]) compared to studies conducted among participants not initiated on ART (34.1 [95% CI 9.8; 71.1]) (Table 3).

The pooled prevalence was higher in studies conducted between 2000–2012 (34.1% [95% CI 15.2;59.9]) compared to studies conducted between 2013–2022 (20.0% [95% CI 9.8;36.5]) (Table 3, Fig. 5). Based on diagnostic criteria, studies that used BP only measurement had a pooled prevalence of 26.7% [95% CI 11.5;50.5], while in studies those that used BP measurement or antihypertensive drug use, the prevalence was 20.7% [95% CI 12.2;32.9], and the two studies that used the self-report criteria yielded a pooled prevalence of 45.8% [95% CI 0.0;100] (Table 3).

### Prevalence of T2D among PLHIV in South Africa

In total, data from 18,555 PLHIV were included from the 18 studies that reported on prevalence of T2D. The diagnostic criteria used to categorise participants as having diabetes varied widely across the studies and included: participant self‐reported history of diagnosed T2D by a professional physician (*n* = 1); self-reported use of insulin or oral hypoglycemic medications (*n* = 1); fasting blood glucose (FBG) ≥ 7.0 mmol/L (*n* = 1); a random blood glucose level ≥ 11.1 mmol/L (*n* = 1); oral glucose tolerance test (OGTT) ≥ 200mg/dL (*n* = 2); HbA1c ≥ 6.5% (*n* = 5); either self-reported use of insulin or FBG (*n* = 2); either self-report or OGTT or FBG or HbA1c (*n* = 1); either non FBG or FBG or medication use (*n* = 1); either medication use or HbA1c ≥ 6.5% (*n* = 1). Two studies did not explicitly report the diagnostic criteria used. Most of the studies that reported prevalence of T2D were conducted in an urban setting (*n* = 9).

**Fig. 8:**
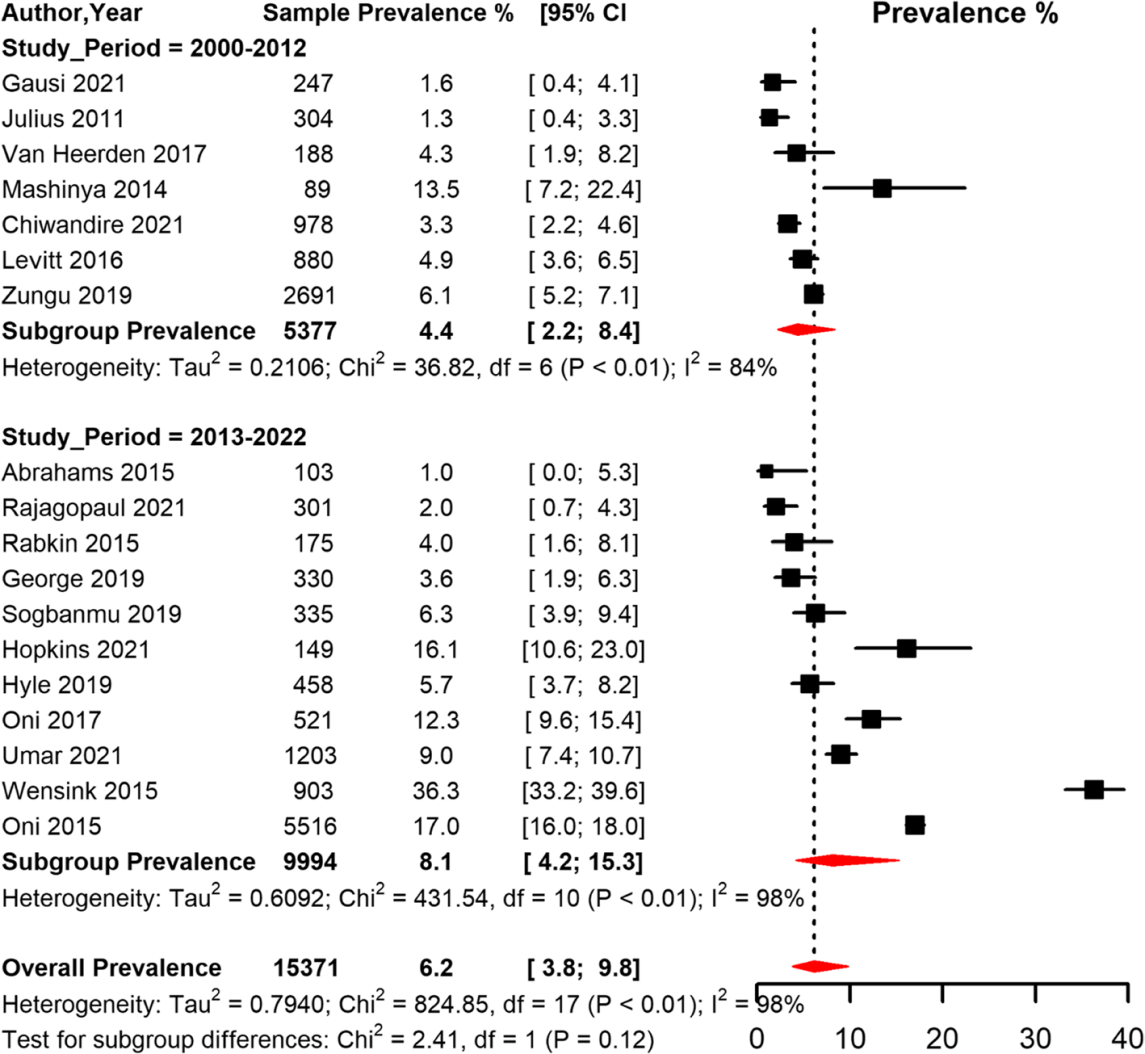
Meta-analysis of prevalence of Type 2 diabetes among people living with HIV in South Africa grouped by study period (2000–2012 and 2013–2022) CI: confidence interval. The vertical dotted line represents the overall prevalence, and the red diagonals represent the pooled prevalence for each group

**Fig. 9:**
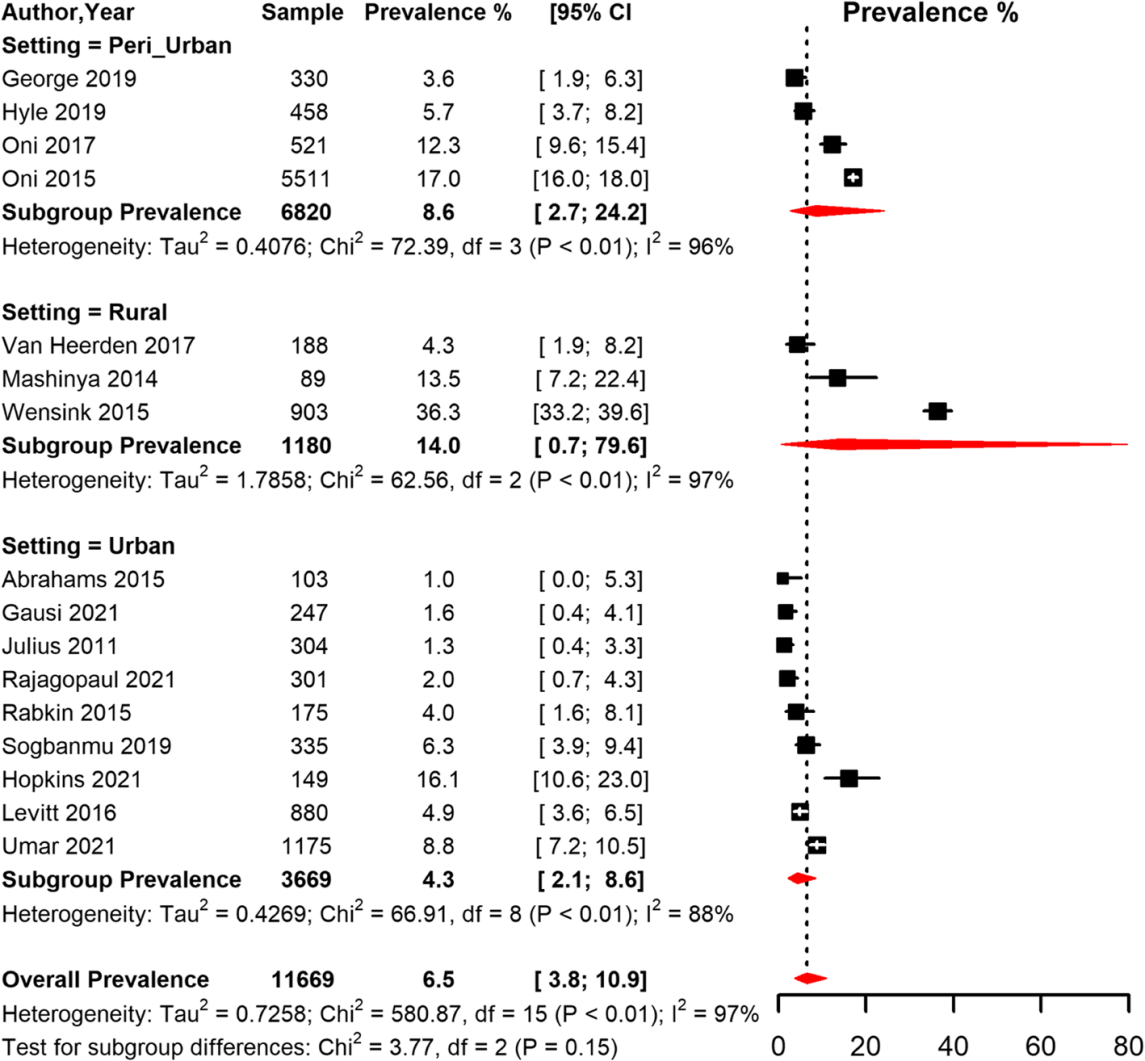
Meta-analysis of prevalence of Type 2 diabetes among people living with HIV in South Africa grouped by setting (rural, peri-urban, and urban areas) CI: confidence interval. The vertical dotted line represents the overall prevalence, and the red diagonals represent the pooled prevalence in each setting

**Fig. 10:**
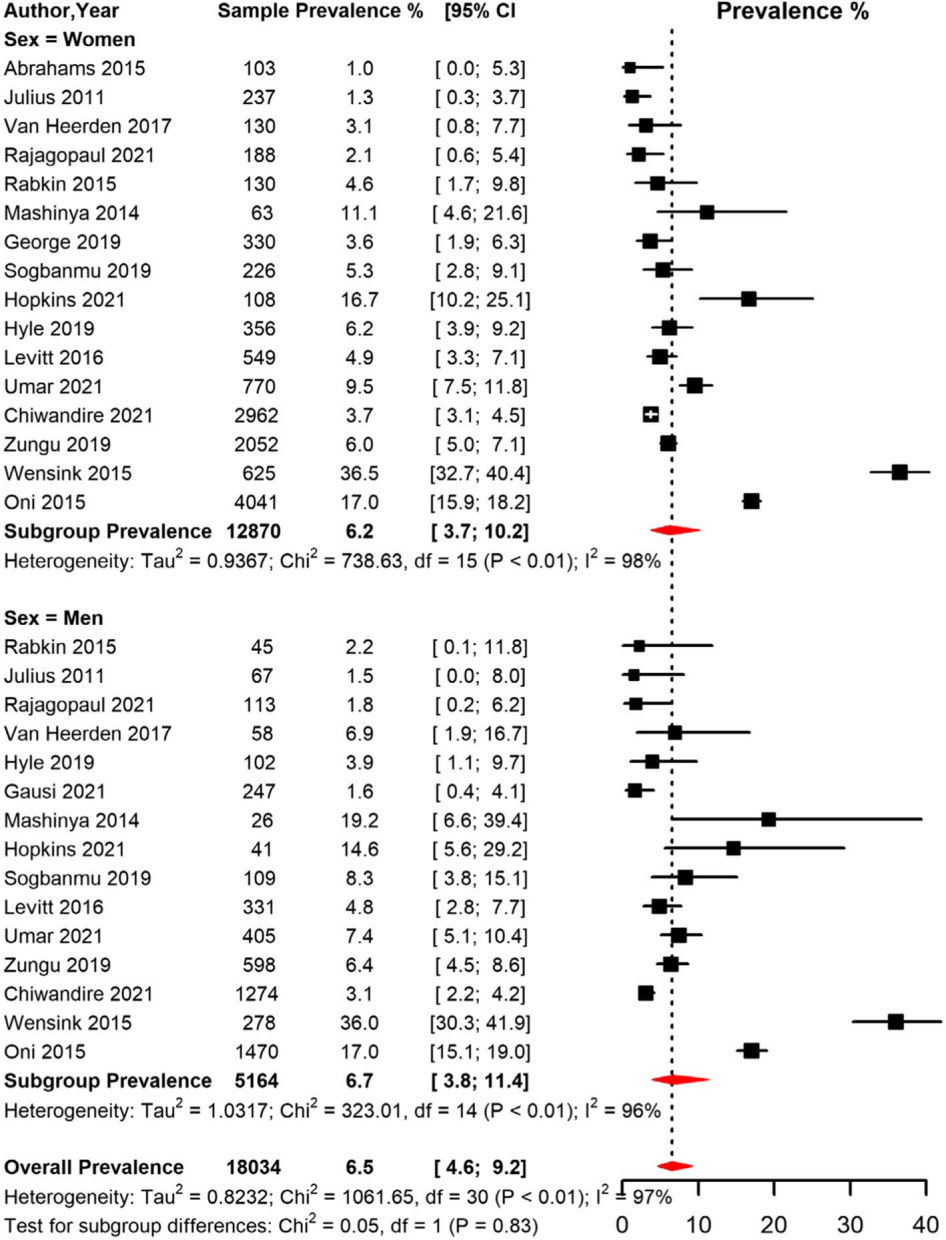
Meta-analysis of prevalence of Type 2 diabetes among people living with HIV in South Africa grouped by sex. CI: confidence interval. The vertical dotted line represents the overall prevalence, and the red diagonals represent the pooled prevalence for each sex

The overall prevalence of T2D among PLHIV in South Africa was 6.1% [95% CI 3.8; 9.7] with high heterogeneity (I^2^ = 99.0%, *p* < 0.001) (Table 4, Fig. 8). The funnel plot (Supplementary Fig. 3) suggested publication bias confirmed by the Egger’s regression test (*p* = 0.011). The sensitivity analysis including only low risk of bias studies yielded a prevalence estimate of 8.0% [95% CI 4,5;13.8] (Table 4). In the subgroup analysis, the pooled prevalence was higher in studies conducted in rural areas (14.0% [95% CI 0.7;79.6]), compared to peri-urban and urban areas (Table 4, Fig. 9). However, the prevalence did not differ significantly by study area. When studies were grouped based on the period when they were conducted, the prevalence was higher in studies conducted between 2013 and 2022 (8.1% [95% CI 4.1; 15.2]) compared to studies conducted between 2000 and 2012 (4.4% [95% CI 2.3; 8.4]) (Table 4, Fig. 8). There was no difference in T2D prevalence between men and women and based on ART status (Table 4, Fig. 10). Further, the pooled prevalence didn’t differ based on the diagnostic criteria.

**Table 4:** Meta-analysis prevalence of Type 2 diabetes among people living with HIV in South Africa

	**prevalence (95% CI)**	**N studies**	**N participants**	**sub-group *p*-value**
**Global** ^Ϣ^	6.1 (3.78;9.73)	18	18,555	
**low ROB** ^β^	8.0 (4.48;13.75)	13	16,565	
**By sex**				
**Males**	6.7 (3.84;11.44)	15	5,164	0.826
**Females**	6.2 (3.72;10.17)	16	12,870	
**By study period**				
**2000–2012**	4.4 (2.31;8.35	7	8,594	0.127
**2013–2022**	8.12 (4.14;15.23)	11	9,961	
**By setting**				
**Rural**	14.0 (0.67;79.62)	3	1,180	0.152
**Urban**	4.3 (2.15;8.56)	9	3,669	
** Peri-urban**	8.60 (2.70;24.16)	4	6,820	
**Diagnostic criteria**				
** HbA1c > = 6.5%**	6.5 (2.33;16.85)	5	2,081	0.718
** OGTT**	5.7 (0.39;49.01)	3	1,504	
**FBG/non FBG** ^a^	7.8 (0.00;99.56)	2	277	
**Insulin/FBG/HbA1c** ^b^	7.7 (1.19;36.43)	5	7,477	
**Others** ^c^	4.4 (2.00;9.43)	3	7,216	
**ART status** ^ϱ^				
**initiated**	5.8 (2.80;11.74)	12	10,536	0.442
**uninitiated**	7.9 (4.30;13.98)	5	3,783	

ART: anti-retroviral therapy; CI: confidence interval; FBG: fasting blood glucose; HBA1c: hemoglobin A1c; OGTT: oral glucose tolerance test; ROB: risk of bias

Ϣ overall pooled prevalence

β pooled prevalence with only studies with a low ROB

^a^ Combined studies that used either FBG or non FBG

^b^ combined studies that used either insulin/medication use, or mixed methods such as medication use and HBA1c, medication use and FBG, medication use and non FBG

^c^ included studies that used self-report of previous physician diagnosis or studies that never reported criteria used

ϱ Initiated means participants were already on ART at time of enrollment into the study while uninitiated means participants were not on ART or initiated ART at start or during course of the study

## Discussion

This systematic review and meta-analysis focused on the syndemics of obesity, HPT and/or T2D with HIV, describing the prevalence of these three NCDs in PLHIV in South Africa. Overall, the prevalence of HPT was highest among PLHIV followed closely by obesity and then T2D. Obesity was higher in PLHIV from urban and peri-urban areas compared to rural areas, while the prevalence of HPT and T2D were higher among PLHIV in rural and peri-urban areas compared to urban areas. The prevalence of obesity and T2D have both increased over time while the prevalence of HPT has decreased over time. Generally, our findings represent pooled estimates of the burden of obesity, HPT, and T2D in PLHIV in South Africa factoring in differences in study setting, period when the studies were conducted, ART status, sex of the participants and diagnostic criteria.

### Obesity

The pooled prevalence of obesity in 13,779 PLHIV from 19 studies was 23.2% with women having a higher prevalence (23.6%) compared to men (12.1%). Our findings are lower than the prevalence estimates that have been reported in the general population of South Africa women (41%) but similar to the men (11%) [58]. Furthermore, our findings agree with the obesity prevalence estimates that have been reported among men and women living with HIV in other low middle income countries (LMIC) in SSA such as in Kenya (women: 22.6%, men: 11.0%) and Nigeria [59–61]. The fact that obesity is still prevalent among PLHIV may be explained by the success of widespread use of ART in South Africa with approximately 4.6 million adults on ART [5]. This is partially supported by the finding reported in this review indicating that participants initiated on ART had a higher prevalence of obesity compared to participants not initiated on ART. ART is reported to be associated with weight gain among PLHIV and evidence of the dual occurrence of obesity and HIV epidemics is increasing in the literature [62–66]. For instance, dolutegravir (DTG) has been reported to impact weight and glucose metabolism among PLHIV [67, 68]. Further, the high burden of obesity in this review is suggestive of co-existence of chronic NCDs with chronic infectious diseases and may reflect an epidemiological transition in the South African population. Moreover, elevated rates of obesity are indicative of increased risk of Cardiovascular diseases, T2D, and musculoskeletal disorders [69].

Our findings indicate that the prevalence of obesity among PLHIV did not differ significantly based on study area, with estimates being relatively similar in urban, peri-urban, and rural areas. This could be attributed to the overall changes in lifestyle and nutritional transition, which are associated with increased access to energy-dense foods, animal-source foods, sugar-sweetened beverages, and less strenuous jobs [66, 70, 71]. However, shifts in the diet are more evident among people residing in urban settings compared to those in rural settings because of higher income and easy access to processed foods [11, 71]. Furthermore, our combined estimates indicated that the prevalence in studies conducted from 2013 to 2022 was lower than that in studies conducted between 2000 and 2012. These findings contradict the reported increase in prevalence in South Africa and SSA as a whole [11]. It's important to note that our results lacked statistical significance and may have been influenced by the substantial heterogeneity in the included studies.

### Hypertension

In this meta-analysis, 22 studies with 123,951 PLHIV reported on the prevalence of HPT, and the pooled prevalence was 25.5%. When analyzed by sex, the prevalence estimates among women (27.0%) and men (22.4%) living with HIV were lower than that reported in the 2016 Demographic Health Survey (DHS) where the HPT prevalence was 46.0% among women and 44.0% among men in the general population of South Africa [58]. Variations could be explained by the diverse diagnostic criteria that were used by the studies included in this review, that involved both objective measurements and participant recall. The 2016 DHS classified HPT using objective measurements (Blood pressure WHO cutoff values). HPT is significantly underdiagnosed in the general population of South African [72, 73] and among PLHIV [29]. Further, limited integration of HIV programs with NCD care during the period when most included studies were conducted could be a barrier to precise ascertainment of HPT burden among PLHIV in South Africa.

In other studies, conducted globally and in SSA, the prevalence of HPT has been shown to be high among PLHIV compared to people not infected with HIV [74, 75]. The most recent meta-analysis on the global estimates of HPT prevalence among PLHIV involving 194 studies from 61 countries with data collected between 2007 and 2018 reported a global pooled prevalence of 23.6% [95% CI 21.6%; 25.5%] and 19.9% [95% CI 17.2; 22.8%] in the Southern and Eastern region of Africa [76]. Our findings correspond to the estimates in SSA and in the Southern African region. In a systematic review by Nguyen et al., [77], the prevalence of HPT was reported to vary widely (ranging from 8.7% to 45.9%) among PLHIV in LMICs, concluding that the burden of HPT among PHLIV is not well established in LMIC. Future research which allows for accurate estimates of diagnosed HPT prevalence among PLHIV in South Africa are necessary to guide health policies for effective management of the dual burden of HIV and HPT.

Our finding showed that the prevalence of HPT among PLHIV did not differ significantly based on area, however rural and peri-urban areas had a higher prevalence compared to urban areas. Similar findings have been reported in studies conducted to estimate burden of HPT in the general South Africa population [19, 78]. In contrast Addo et al., [79], reported the majority of hypertensive patients to be urban dwellers in SSA. Based on our findings, there is a need to develop and intensify existing healthcare systems in rural areas and peri-urban townships without overlooking urban areas to optimize HPT care especially among PLHIV.

### Type 2 diabetes

The overall prevalence of T2D in 18 studies with 18,555 PLHIV was 6.1% with high heterogeneity between the individual studies. Stratified by sex, our findings showed that the prevalence was similar in both men and women (6.7% and 6.2% respectively).Studies such as a meta-analysis by Pheiffer et al.,[21], a survey study by Egede et al. [80], and a study by Grundlingh et al. [81], reported varying prevalence estimates of 15.3%, 1.7% and 22% respectively in the general South African population. The difference in the findings may be attributed to wide diversity of study populations, differences in the participants’ lifestyles and a wide variety of diagnostic criteria used. For instance, in this review, different criteria were used to diagnose T2D such as FBG, OGTT, HbA1c, non FBG, insulin use and self-report, hence resulting in varying estimates of T2D burden. OGTT is the recommended criterion for diagnosis of T2D in Africa [82]. Standardisation of the diagnostic criteria will help address issue of variation in diagnoses. Our reported overall prevalence estimates contrast to studies conducted in different parts of SSA [83–85]. Variations in estimates may be partly attributed to challenges with proper diagnosis of T2D in PLHIV in South Africa, whereby a significant proportion of patients are underdiagnosed [81, 86]. With the increasing T2D related risks such as rapid urbanisation, physical inactivity, and unhealthy diets in South Africa, continued monitoring and tracking of T2D at both population and individual levels mostly among PLHIV is necessary.

Our findings show that the pooled prevalence of T2D among PLHIV was higher in rural (14.0%), and peri-urban (8.6%) settings compared to urban settings (4.3%). Though unsignificant, these findings are contrary to other results reported in South Africa and SSA where the prevalence was reported to be higher in urban settings [20, 87]. Because of the existing association between HIV infection and increased risk of T2D [88, 89], limited access to healthcare, and high unemployment in both urban and rural settings of South Africa, there is need for integration of screening, monitoring and interventions for T2D into HIV programs.

The pooled prevalence of T2D was higher in studies conducted between 2013 and 2022 compared to 2000 and 2012. The results, which demonstrate an increase in T2D in more recent times, agree with those reported in previous studies, systematic reviews, and meta-analyses [74, 76, 89]. This could be explained by increased life expectancy among PLHIV [90] and epidemiological transitions in South Africa such as population growth and aging [91, 92]. In addition, ART coverage has improved in South Africa and globally resulting in longer life and reduced mortality rates among PLHIV [6, 90]. Also, the national ART treatment guidelines have been changing ART roll-out in SA, for instance in 2009, ART guidelines moved from monotherapy to dual therapy [93], this may be a salient factor influencing the observed trends in co-morbidities between the older papers and more recent ones. Furthermore, the elevated estimates of T2D could also be due to an increasing burden of obesity, increasing urbanization, adaptation of lifestyle behaviors and their interaction with the genetic predisposition to T2D within the South African population [94, 95].

### Implication of the findings

The findings in this meta-analysis have implications on practice and further research of syndemics of NCDs and HIV in South Africa and other LMICs with high burden of HIV. The high and varying prevalence of obesity, HPT and T2D in PLHIV highlights the need for scaling of the South Africa national government’s plan for integrated chronic disease management. Integration of NCD services such as screening, diagnosis, prevention (primary and secondary) and treatment into HIV programs has been shown to be beneficial in addressing the increasing dual burden of HIV and cardiovascular diseases risk factors (obesity, T2D, and HPT) [7, 96–98]. Due to the high burden of HIV, South African health care system should be strengthened to face the increasing dual burden of NCDs and HIV.

### Limitations

This meta-analysis is not without limitations. Firstly, the review included some studies with moderate risk of bias; although after considering only studies with low risk of bias, the overall estimates were similar to the crude analysis estimates. Secondly, there was high heterogeneity between the studies, an issue common to meta-analysis of prevalence studies, therefore the pooled estimates need to be interpreted with caution. Higher heterogeneity increases uncertainty in the pooled estimates and reduces generalizability of the results. Sub-group analyses, based on settings (rural, urban, peri-urban), sex, ART status, diagnostic criteria, and study year, were conducted to pinpoint sources of heterogeneity, though the issue persisted. Notably, some other variables that could have contributed to the observed heterogeneity in the original studies such as CD4 cell count, HIV viral load, duration on ART, ART regimen and sociodemographic factors were not explicitly reported. Additionally, some subgroup analyses were not statistically significant hence it’s not definitive that the prevalence estimate for one subgroup is higher or lower compared to another. Thirdly, caution is needed when using obesity estimates, as many studies relied solely on BMI, which may not be suitable due to factors such as genetic diversity and sociocultural factors in Sub-Saharan Africa. Complementary assessments like waist circumference, body composition, and metabolic risk factors offer a more comprehensive assessment. Finally, the provinces were variably represented with more studies in WC, KN and GA provinces compared to other provinces in South Africa. Hence weakening generalizability of our findings and requiring that more studies on NCDs among PLHIV are conducted in NC, EC, LM, FS, NW and MP provinces. Irrespective of these limitations, this meta-analysis provided a clear summary of the existing evidence on the burden of obesity, HPT and T2D among PLHIV in South Africa according to setting (rural, peri-urban, or urban area), sex, and how the burden has varied over time in the era of increased life expectancy among PLHIV.

## Conclusion

We found that among PLHIV in South Africa, the prevalence of HPT was highest, closely followed by obesity, and then T2D. The pooled prevalence estimates for obesity and HPT were higher in peri-urban areas, whereas the pooled prevalence of T2D was higher in rural areas. The prevalence estimates of obesity and T2D have increased over time. These findings add to the already existing literature about the increasing burden of cardiovascular disease risk factors, specifically obesity, HPT and T2D in LMIC with a high burden of HIV. However, caution needs to be taken while interpreting these results because of heterogeneity in the in studies. More research focusing on understanding syndemics of NCDs, and HIV epidemics is needed particularly in setting with a high burden of HIV. This would then guide enhancement of cost-effective interventions that involve integrated HIV and NCDs care such as screening, diagnosis, prevention, and treatment programs in LMIC faced with a dual burden of HIV and NCD epidemics.

### Supplementary Information

**Additional file 1.**

**Additional file 2.**

## Data Availability

All data analyzed during this study are included in this published article [and its supplementary information files].
