# Supplementary material for: Prevalence of obesity, hypertension and diabetes among people living with HIV in South Africa: a systematic review and meta-analysis
Source: BMC Infect Dis. 2023 Dec 7;23:861. doi: 10.1186/s12879-023-08736-5 (PMC10704741; doi:10.1186/s12879-023-08736-5)

Supplementary figure 1: Funnel plot of the prevalence of obesity among PLHIV in South Africa

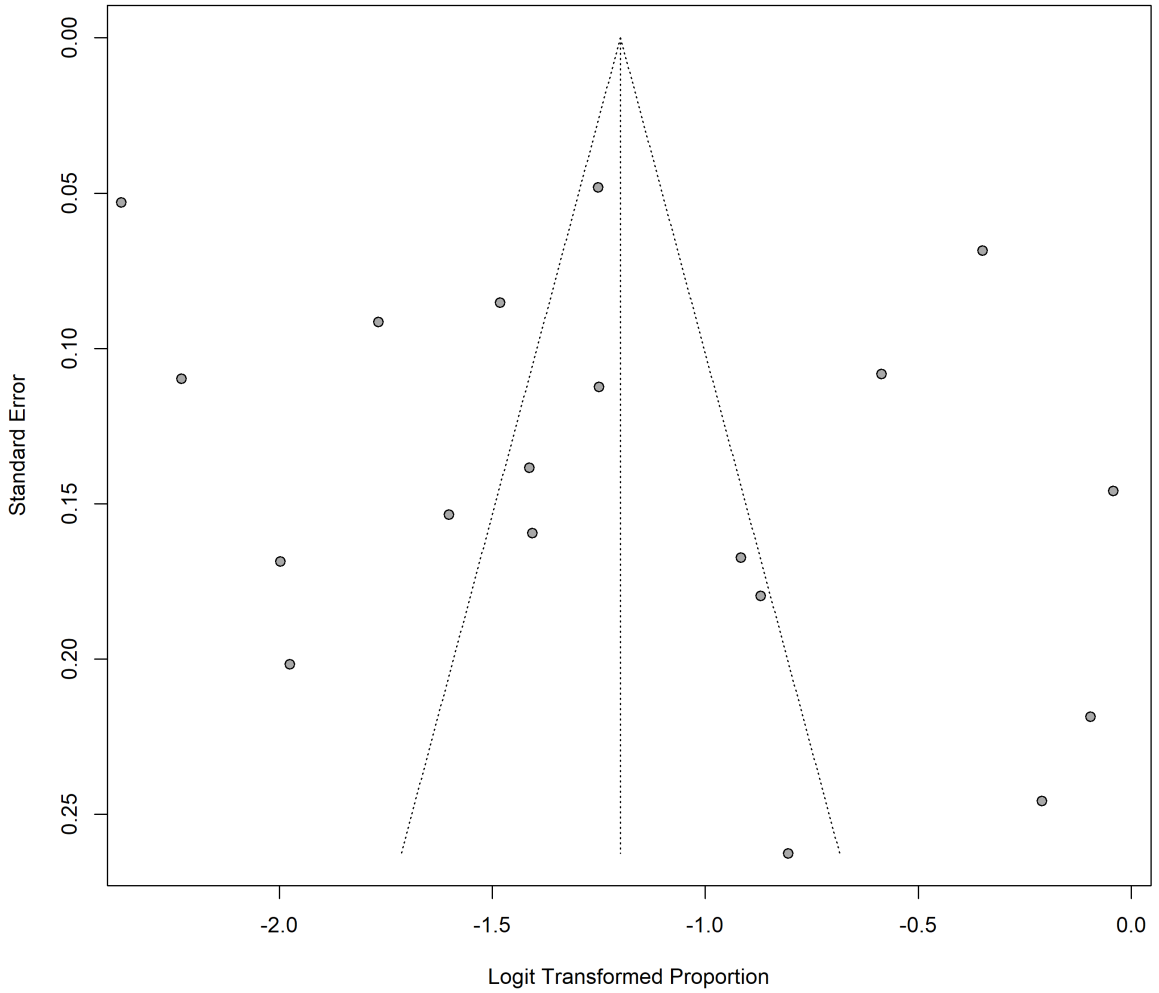

Supplementary figure 2: Funnel plot of the prevalence of HPT among PLHIV in South Africa

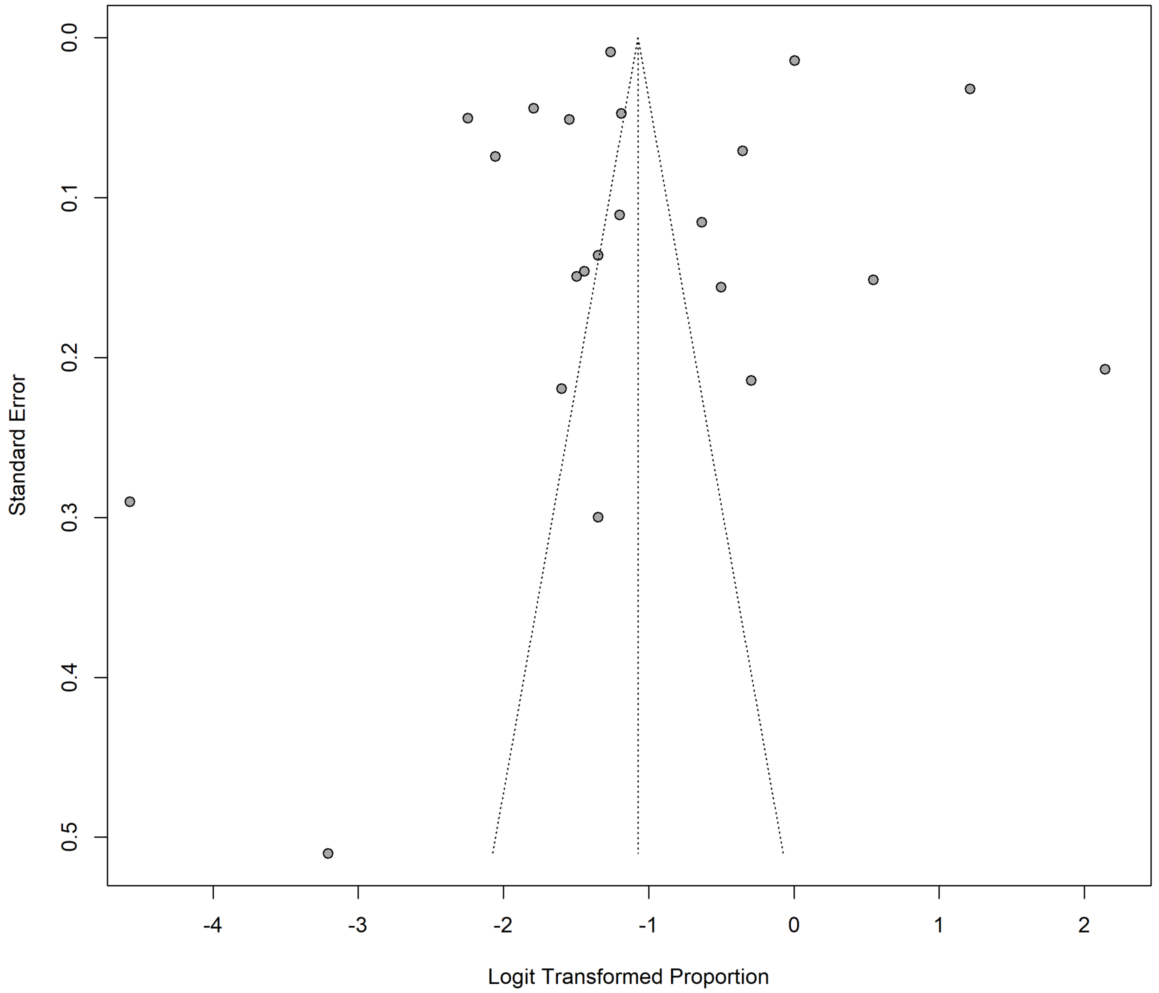

Supplementary figure 3: Funnel plot of the prevalence of T2D among PLHIV in South Africa

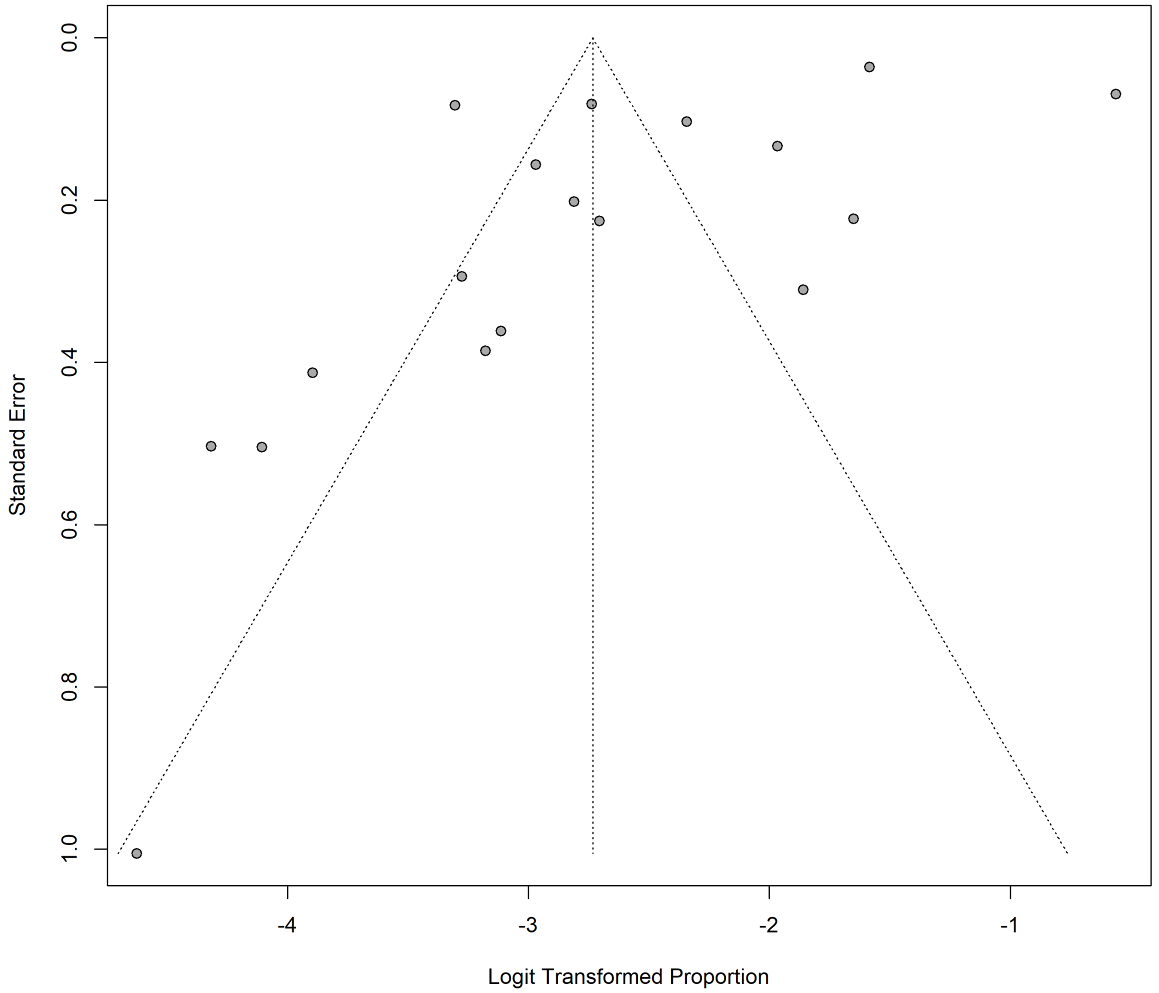

Supplement: Supplementary file 1 — Additional file 1. [file 12879_2023_8736_MOESM1_ESM.pdf]
