# Supplementary material for: Prevalence of obesity, hypertension and diabetes among people living with HIV in South Africa: a systematic review and meta-analysis
Source: BMC Infect Dis. 2023 Dec 7;23:861. doi: 10.1186/s12879-023-08736-5 (PMC10704741; doi:10.1186/s12879-023-08736-5)
Supplement: Supplementary file 2 — Additional file 2. [file 12879_2023_8736_MOESM2_ESM.pdf]

Supplementary table 1: PubMed Search Strategy

#	search query	Results
**1**	Search: ((HIV) OR (people living with HIV)) OR (PLHIV)	398,184
**2**	Search: ((prevalence) OR (epidemiology)) OR (burden)	3,544,956
**3**	Search: ((((((obesity) OR (obese)) OR (hypertension)) OR (high blood pressure)) OR (type 2 diabetes)) OR (T2D)) OR (diabetes mellitus)	1,530,335
**4**	Search: (((((((south africa) OR (western cape)) OR (KwaZulu natal)) OR (Limpopo)) OR (Mpumalanga)) OR (free state)) OR (eastern cape)) OR (Gauteng)	460,28
**5**	#1 AND #2	132,216
**6**	#5 AND #3	4,347
**7**	#6 AND #4	720
